# Effects of Short-term Renovascular Hypertension and Type 2 Diabetes on Cardiac Functions in Rats

**Published:** 2014-01

**Authors:** Ali Akbar Nekooeian, Azadeh Khalili, Mohammad Bagher Khosravi

**Affiliations:** 1 Department of Pharmacology, School of Medicine, Shiraz University of Medical Sciences, Shiraz, Iran;; 2 Cardiovascular Pharmacology Research Center, School of Medicine, Shiraz University of Medical Sciences, Shiraz, Iran;; 3 Department of Anesthesiology, School of Medicine, Shiraz University of Medical Sciences, Shiraz, Iran

**Keywords:** Renovascular hypertension, •Type 2 diabetes mellitus, Cardiac functions

## Abstract

**Background: **The cardiac effects simultaneously occurring during experimental hypertension and diabetes have rarely been investigated. This study aimed at examining the effects of short-term renovascular hypertension and type 2 diabetes on cardiac functions.

**Methods: **Five groups (7 each) of male Sprague-Dawley rats, including a control group, a diabetes (induced by Streptozocin and Nicotinamide) group, a renovascular hypertensive (induced by placing Plexiglas clips on the left renal arteries) group, a sham group, and a simultaneously hypertensive-diabetic group, were used. The animals’ hearts were used for isolated heart studies, and the indices of cardiac functions and coronary effluent creatine kinase MB were measured. The results were analyzed using One-way Analysis of Variance, followed by the Duncan Multiple Range test.

**Results:** The diabetic group had a significantly lower rate of rise (-29.5%) and decrease (-36.18%) in ventricular pressure, left ventricular developed pressure (-28.8%), and rate pressure product (-35%), and significantly higher creatine kinase MB (+166%) and infarct size (+36.2%) than those of the control group. The hypertensive group had a significantly higher rate of rise (+12.17%) and decrease (+16.2%) in ventricular pressure, left ventricular developed pressure (+16%), and rate pressure product (+24%), and significantly lower creatine kinase MB (-30%) and infarct size (-27%) than those of the sham group. Simultaneously, the diabetic and hypertensive rats had a significantly higher rate of rise (+32%) and decrease (+30.2%) in ventricular pressure, left ventricular developed pressure (+17.2%), and rate pressure product (+22.2%), and significantly lower creatine kinase MB (-24%) and infarct size (-16.2%) than those of the diabetic group.

**Conclusion: **The findings indicated that the simultaneity of hypertension with type 2 diabetes attenuated diabetes-induced cardiac impairment.

## Introduction

Experimental models of hypertension and diabetes type 2 indicate that such diseases are associated with changes in cardiac functions. It has been shown that diabetes is associated with impaired as well as improved cardiac functions. Hearts isolated from experimental models of diabetes, induced by either Streptozotocin (STZ) or Alloxan, exhibited severe impaired functions manifested by higher infarct size and mortality following ischemia and reperfusion,^1^^-^^2^ lower coronary flow,^3^ higher coronary resistance,^4^ lower left ventricular developed pressure (LVDP),^3^ and lower cardiac power.^5^ On the other hand, experimental diabetes was associated with improved cardiac function, characterized by higher rate pressure product (RPP), LVDP, and lower release of creatine kinase MB (CK-MB) during reperfusion.^6^

There is no agreement on the cardiac effects of experimental hypertension. Spontaneous hypertension in rats does not change^7^ or increase^8^the indices of cardiac contractility. Furthermore, experimental hypertension is associated with higher infarct size and probability of arrhythmia following ischemia reperfusion,^9^ decreased recovery of LVDP,^8^ and higher coronary resistance.^8^

It is generally believed that hypertension enhances the cardiovascular effects of diabetes. Whether or not such a generalization remains true at every stage of the diseases has not been examined. A few published studies have indicated that hearts form diabetic hypertensive animals may be less protected.^8^^,^^10^ Moreover, hypertension deteriorates the cardiovascular complications of diabetes, and the complications of simultaneous hypertension and diabetes were more severe than those of either hypertension or diabetes.^8^ There is; however, no experimental information on the effects of type 2 diabetes and renovascular hypertension on cardiac functions. Therefore, the present study was designed to examine the effects of experimental short-term renovascular hypertension on cardiac functions in type 2 diabetes in rats using the Langendorff technique.

## Materials and Methods

*Animals*

Male Sprague-Dawley rats (200-250 g) were obtained from the Laboratory Animal Breeding Center, Shiraz University of Medical Sciences, Shiraz, Iran. They were kept under standard conditions (light/dark cycle: 12 h; humidity: 25-35%; and temperature: 22-28˚C) with standard food and water ad libitum. All the animal procedures were approved by the Institutional Committee for Care and Use of Animals. 

*Materials*

Streptozotocin was obtained from Teva Parenteral Medicine Inc. (Irvine, CA, USA). Nicotinamide (NA) was purchased from Sigma-Aldrich Chemical Co. (Steinheim, Germany). Streptozotocin and NA were dissolved in sodium chloride (0.9%). Ketamine and Xylazine were obtained from Alfasan (Woerden, Holland). Triphenyltetrazolium Chloride (TTC) was obtained from Sigma-Aldrich Chemical Co. (Steinheim, Germany). Kits for the measurement of coronary artery effluent creatine kinase (CK-MB) were obtained from Pars Azmoon Company (Pars Azmoon, Tehran, Iran).

*Experimental Design*

The animals (n=35) were randomly allocated to five groups, including a type 2 diabetes control group (n=7), a type 2 diabetes group (n=7), a renovascular hypertensive (HTN) group (n=7), a sham-operated group (Sham) (n=7) as a control for the renovascular hypertensive group, and a simultaneous type 2 diabetes (n=7) and renovascular hypertensive (HTN) group (n=7).

*Experimental Protocol*

*Induction of Diabetes and Renovascular Hypertension*

Type 2 diabetes was induced by injecting the animals with single intraperitoneal administrations of NA (110 mg/kg) 15 min before single intraperitoneal administrations of STZ (60 mg/kg).^11^ Seven days later, the animals’ blood glucose was determined using a Glucometer (Accu-Chek^®^ active, Mannheim, Germany), and those with fasting blood glucose (FBG) levels higher than 126 mg/dl were taken as having type 2 diabetes.^12^ Six weeks after the injection of NA and STZ or normal saline, the animals were subjected to sham operation or placement of solid Plexiglas clips on the left renal arteries to induce two-kidney, one-clip renovascular hypertension as was described previously.^13^ Briefly, under Ketamine (60 mg/kg) and Xylazine (8 mg/kg) anaesthesia, incisions were made in the left flanks, and the left renal arteries and veins were exposed. The arteries were gently dissected from the veins, and solid Plexiglas clips (internal diameter=0.20-0.22 mm) were placed on the left renal arteries. The abdominal wall and skin incisions were then sutured using absorbable (catgut-3/0) and non-absorbable (silk-3/0) suture materials, respectively. The sham-operated animals were subjected to the same procedure, but no clip was placed on the left renal arteries.^13^

Four weeks after the sham-operation or induction of renovascular hypertension, the animals’ systolic blood pressure (SBP) and heart rate (HR) were measured using the noninvasive tail-cuff (Chart 5.0 software, PowerLab 4/30, ADInstruments Inc., MA, Sydney, Australia) method. Three consecutive measurements of blood pressure, which had a difference of less than 5 mm Hg, were considered valid. The mean of these three measurements were recorded as a valid value of SBP on every occasion. The animals that had systolic blood pressure >160 mm Hg were considered hypertensive.^13^ The animals were then anaesthetized with Ketamine (60 mg/kg) and Xylazine (8 mg/kg), and blood samples were obtained for the measurement of FBG. Afterwards, the animals were sacrificed and their hearts were used for isolated (Langendorff) studies.

*Isolated Heart Study*

The animals’ hearts were mounted, via aorta, on a Langendorff apparatus (ADInstruments, model: LE05200, PanLab, Spain), and perfused retrogradely with Krebs-Henseleit buffer with a pH of 7.4 and following composition in mmol/L: NaCl 118.0; KCl 4.7; CaCl_2_ 2.5; MgSO_4_ 1.2; KH_2_PO_4_ 1.2; NaHCO_3_ 25.0; and glucose 11.0. The buffer was kept at 37ºC, bubbled constantly with 95 % O_2_ and 5 % CO_2_, and infused at a constant flow. Through the left atrium, a latex balloon was placed in the left ventricle. The balloon’s catheter was connected to a PowerLab 8/30 data acquisition system (Chart 5.0 software, PowerLab 8/30, ADInstruments Inc., MA, Sydney, Australia) via a pressure transducer for continuous recording of the cardiac function. The balloon was then inflated to an end-diastolic pressure of 5-10 mm Hg. The Langendorff mode was switched to constant-pressure (60 mm Hg) for the rest of the experiment.

The mounted hearts were allowed to equilibrate for 30 min, and a baseline measurement of left ventricular systolic pressure (LSVP), LVEDP, +dp/dt, -dp/dt, and HR was performed. The hearts were then subjected to 20 min global ischemia (zero-flow), followed by a 60 min of reperfusion. Samples of coronary effluent for the measurement of CK-MB were collected in the first minute of reperfusion, and kept frozen (-80ºC) until analysis. The above-mentioned cardiac parameters were measured every 15 min during reperfusion. At the end of reperfusion, cardiac infarct size was determined using TTC staining.^14^

*Determination of Cardiac Infarct Size*

The hearts were cut into 2-mm thick slices, which were incubated in TTC solution (1%) at 37ºC for 20 min. The slices were then incubated with 10% formalin for 24 h. Afterwards, they were digitalized using a digital camera (Powershot G1, Canon, Tokyo, Japan), and the infarct areas were quantified as the percentage of the total area of the slices using an image analysis software (Scion Image pro. 1.16, NIH, USA).^14^

*Biochemical Measurements*

Coronary effluent CK-MB was measured according to the manufacturers’ instructions.

*Calculations and Statistical Analysis*

Left ventricular developed pressure was calculated as LVSP-LVEDP. Rate pressure product was calculated as LVDP×HR. The data, presented as mean±SEM, were compared using the One-way Analysis of Variance (ANOVA), followed by the Duncan Multiple Range test. A P value ≤0.05 was considered statistically significant. Data analysis was performed using SigmaStat statistical software (version 3.0) (San Jose, CA, USA). The illustrations were prepared using SigmaPlot software (version 8.0) (San Jose, CA, USA).

## Results

*Blood Pressure and Heart Rate*

There was no significant difference in the SBP of the type 2 diabetes control, type 2 diabetes, and sham groups at 4 weeks after the sham-operation (table 1). The SBP of the HTN group was significantly higher than those of the sham and type 2 diabetes groups. Moreover, the SBP of the type 2 diabetes+HTN group was significantly higher than that of the HTN group. The HR of the type 2 diabetes group at 4 weeks after the sham operation was significantly lower than that of the relevant control group (table 1). Moreover, the HR of the HTN group was significantly higher than that of the sham group. Also, the HR of the type 2 diabetes+HTN group was significantly higher than that of the type 2 diabetes group (table 1).******

**Table 1:** Systolic blood pressure (SBP, mm Hg), heart rate (HR, beats/min), fasting blood glucose (FBS, mg/dl), infarct size (IS, percentage of left ventricle), and concentration of creatine kinase MB (CK-MB, U/ml) in the coronary effluent of the diabetic control (DM2-C), diabetic (DM2), hypertensive control (sham), renal hypertensive (HTN), and simultaneously diabetic hypertensive (DM2+HTN) groups

	**SBP**	**HR**	**FBG**	**CK-MB**	**IS**
DM2-C	117.0±1.30	401.6±4.9	113±04	9.4±1.1	42.2±2.3
DM2	117.8±2.4	373.4±5.0*	213±12*	25.0 ±2.2*	57.5±2.5*
Sham	115.7±1.5	403.2±4.0	116±03	10.0±0.8	41.5±2.3
HTN	174.6±3.0*	453.1±9.5*	124±04	7.0±0.6*	30.4±2.5*
DM2+HTN	183.5± 2.6†	450.0±7.3‡	192±20†	19.0±1.4†‡	48.2±1.0†‡

Data are presented as mean±SEM. *Significant difference (P≤0.05) from the DM2-C or the sham group; †Significant difference (P≤0.05) from the HTN group; ‡Significant difference (P≤0.05) from the DM2 group

*Isolated Heart Studies*

**Figure 1:**
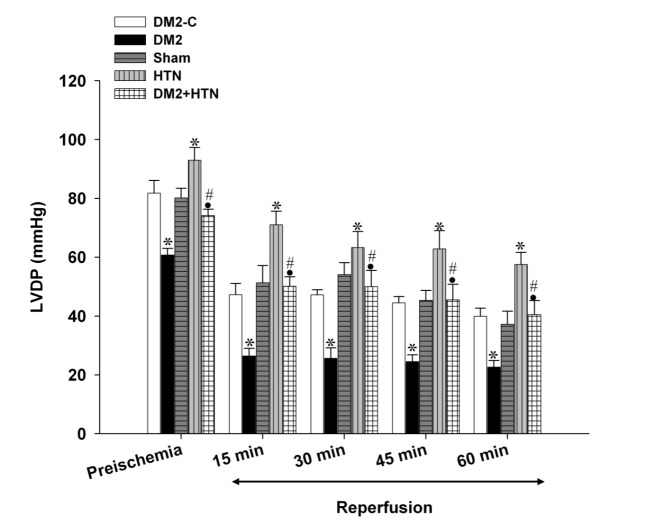
Left ventricular developed pressure (LVDP, mm Hg) of diabetic control (DM2-C), diabetic (DM2), hypertensive control (sham), renal hypertensive (HTN) and simultaneously diabetic hypertensive (DM2+HTN) groups (n=7-8 each) at baseline (pre-ischemia), and at 15, 30, 45, and 60 minutes of reperfusion. Data are presented as mean±SEM. *Significant difference (P≤0.05) from the DM2-C group; #Significant difference (P≤0.05) from the DM2 group; ●Significant difference (P≤0.05) from the HTN group

**Figure 2:**
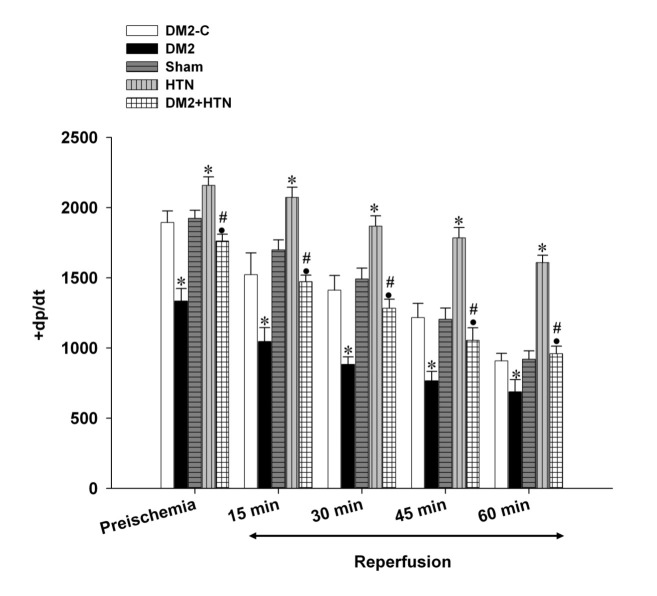
Rate of rise in ventricular pressure (+dp/dt, mm Hg/min) of the diabetic control (DM2-C), diabetic (DM2), hypertensive control (sham), renal hypertensive (HTN), and simultaneously diabetic hypertensive (DM2+HTN) groups (n=7-8 each) at baseline (pre-ischemia), and at 15, 30, 45, and 60 minutes of reperfusion. Data are presented as mean±SEM. *Significant difference (P≤0.05) from the DM2-C group; #Significant difference (P≤0.05) from the DM2 group; ●Significant difference (P≤0.05) from the HTN group

**Figure 3:**
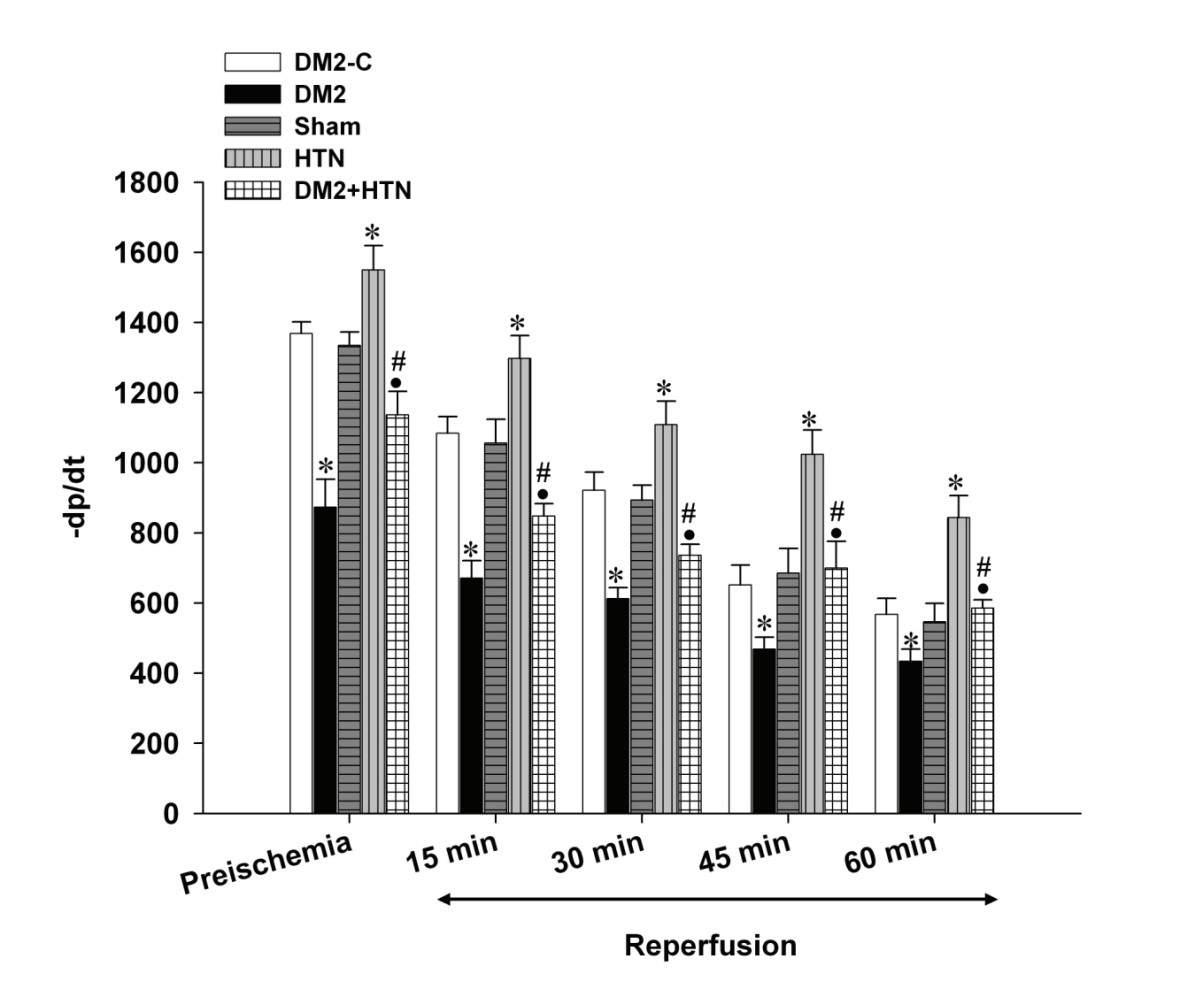
Rate of decrease of ventricular pressure (-dp/dt, mm Hg/min) of the diabetic control (DM2-C), diabetic (DM2), hypertensive control (sham), renal hypertensive (HTN), and simultaneously diabetic hypertensive (DM2+HTN) groups (n=7-8 each) at baseline (pre-ischemia), and at 15, 30, 45, and 60 minutes of reperfusion. Data are presented as mean±SEM. *Significant difference (P≤0.05) from the DM2-C group; #Significant difference (P≤0.05) from the DM2 group; ●Significant difference (P≤0.05) from the HTN group

**Figure 4:**
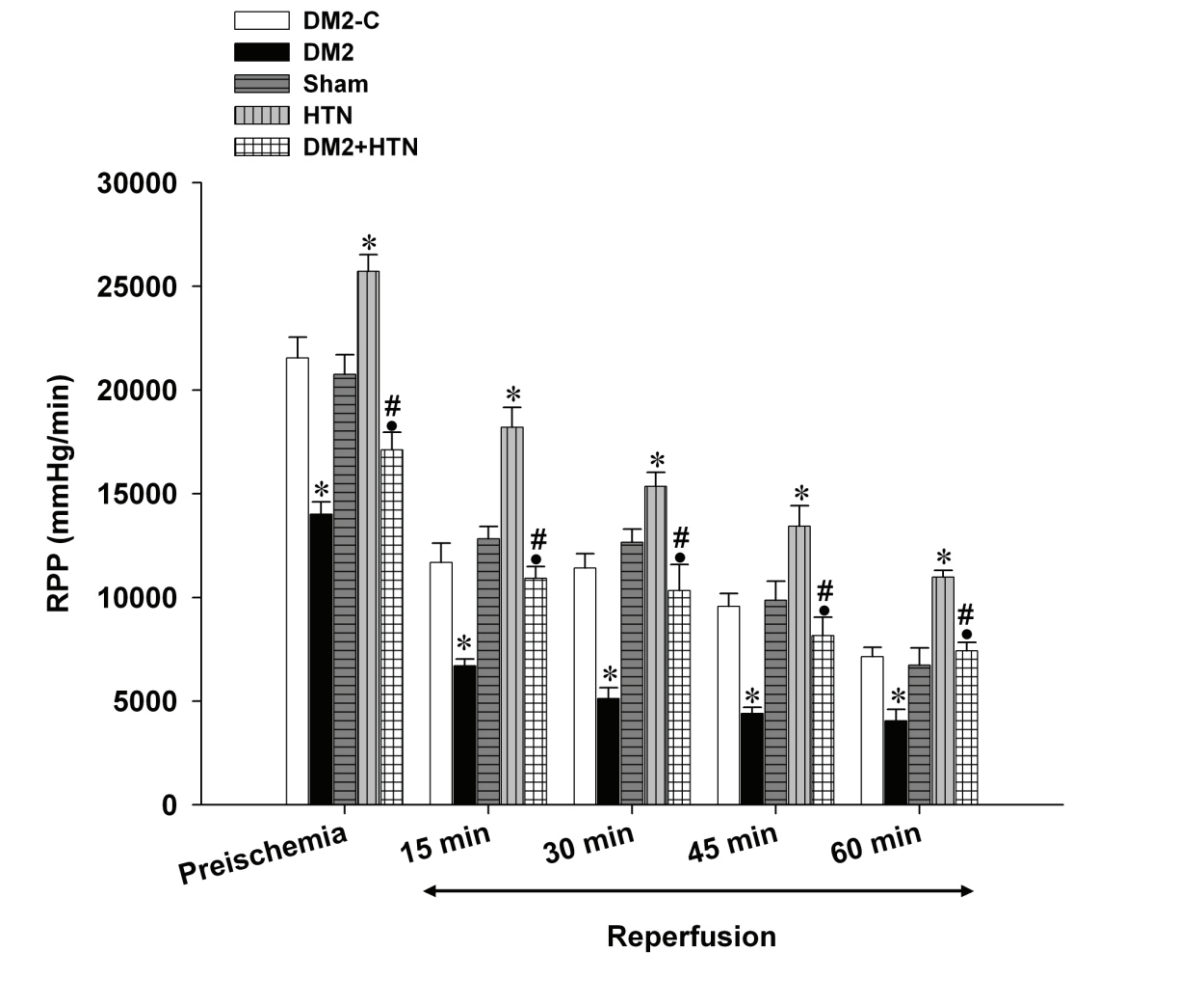
Rate pressure product (mm Hg.beats/min) of the diabetic control (DM2-C), diabetic (DM2), hypertensive control (sham), renal hypertensive (HTN), and simultaneously diabetic hypertensive (DM2+HTN) groups (n=7-8 each) at baseline (pre-ischemia), and at 15, 30, 45, and 60 minutes of reperfusion. Data are presented as mean±SEM. *Significant difference (P≤0.05) from the DM2-C group; #Significant difference (P≤0.05) from the DM2 group; ●Significant difference (P≤0.05) from the HTN group

Pre-ischemic (baseline) LVDP, +dp/dt, -dp/dt, and RPP of the type 2 diabetes rats were significantly lower than those of the relevant control group (figures 1,2,3,4). Moreover, the LVDP, +dp/dt, -dp/dt, and RPP of the HTN group were significantly higher than those of the sham group. In addition, the LVDP, +dp/dt, -dp/dt, and RPP of the type 2 diabetes+HTN group were significantly higher than those of the type 2 diabetes group. A similar pattern of statistical differences was found at 15, 30, 45, and 60 minutes of reperfusion (figures 1-4)

*Biochemical Assays*

There was no significant difference between the FBG (mg/dl) of the sham, type 2 diabetes control, and HTN groups (table 1). However, the FBG of the type 2 diabetes and type 2 diabetes+HTN groups was significantly higher than those of the sham, type 2 diabetes control, or HTN groups. The concentration of CK-MB in the coronary effluent of the type 2 diabetes group was significantly higher than that of the type 2 diabetes control group (table 1). However, the concentration of CK-MB of the HTN group was significantly lower than that of the sham group. The levels of the CK-MB of the type 2 diabetes+HTN group were significantly higher and lower than those of the HTN and type 2 diabetes groups, respectively.

*Myocardial Infarct Size*

The infarct size of the type 2 diabetes group was significantly higher than that of the type 2 diabetes control group, but the infarct size of the HTN group was significantly lower than that of the sham group (table 1). Moreover, the infarct size of the type 2 diabetes+HTN group was significantly higher and lower than those of the HTN and type 2 diabetes groups, respectively.

## Discussion

The main objective of the present study was to examine the effects of simultaneous short-term renovascular hypertension and experimental type 2 diabetes on rat cardiac functions using the Langendorff technique. Our results revealed that short-term renovascular hypertension attenuated the diabetes-induced cardiac impairment. 

The findings of the present study also indicated that the present model of experimental type 2 diabetes was associated with impaired cardiac function, characterized by decreased HR, LVDP, +dp/dt, -dp/dt, and RPP as well as with increased infarct size and coronary effluent CK-MB. Such findings are in agreement with those of some other studies^1^^,^^15^^,^^16^ and indicative of cardiomyopathy.^16^^,^^17^

The mechanism of type 2 diabetes-induced cardiac impairment is not clearly known. However, such an impairment has been attributed to defects in Na^+^/H^+^ and Na^+^/Ca^2+^ exchangers,^18^ calcium ion metabolism,^19^ chronic hyperglycemia (which could affect the expression of some specific genes that encode potassium channel proteins), or increased oxidative stress and apoptosis in the myocardial cells.^20^^,^^21^

Our results showed that the present model of short-term renovascular hypertension was associated with improved cardiac function, characterized by increased HR, LVDP, +dp/dt, -dp/dt, and RPP as well as with decreased myocardial infarct size and coronary artery CK-MB. There are no previous reports on the protective effects of short-term two-kidney, one-clip renovascular hypertension on cardiac performance using the Langendorff technique. Moreover, the effects of other models of hypertension on cardiac functions have not been widely investigated, and there is no agreement in the findings of the published studies. Averill et al.^22^ reported that 9-week two-kidney, one-clip hypertension impaired cardiac performance in rats by impairing stroke volume, cardiac output, and stroke work. Moreover, cardiac performances were also lower in 6-week two-kidney, one-clip renovascular hypertensive rats.^23^ Nevertheless, Schunkert ^24^ showed that hypertension, induced by 8-9-week chronic aortic stenosis in rats, was allied with significantly higher LVSP, LVDP, and +dp/dt. In addition, other studies have reported that experimental hypertension is associated with normal^25^ or increased^8^ cardiac contractility. It is tempting to suggest that the cardiac effects of hypertension might be dependent on the duration of hypertension. Accordingly, in the early stages, in which the heart tries to overcome the increased afterload, hypertension might be associated with increased cardiac performance. However, at later stages the hypertension-induced hypertrophy and remodelling may result in the impairment of cardiac functions.

The mechanism of cardioprotection by short-term hypertension is not clear. Nonetheless, it might be due to increased myocardial responsiveness to calcium ion,^19^ increased sympathetic activity,^26^ increased plasma levels of Ang-(1-7)^27^ (believed to be a potent anti-ischemic and cardioprotective agent),^8^ or increased angiogensis.^28^ Decreased infarct size and CK-MB concentration in the coronary effluent in the renal hypertensive group is in agreement with increased angiogenesis in this model. Further studies are required to examine the mechanisms by which short-term hypertension offers cardioprotection.

As far as the literature is concerned, the present study represents the first of its kind to examine the effects of short-term renovascular hypertension on the cardiac effects of experimental type 2 diabetes. The findings indicated that compared to diabetes alone, the simultaneity of short-term renal hypertension with type 2 diabetes was associated with cardioprotection, characterized by improved HR and cardiac hemodynamic parameters as well as reduced myocardial infarct size and coronary artery effluent CK-MB. This suggests that short-term renovascular hypertension prevented type 2 diabetes-induced cardiac impairment. The mechanisms of such an effect are not clear; however, they might be due the above-said mechanism, namely increased myocardial responsiveness to calcium ion,^19^ increased sympathetic activity,^26^ increased plasma levels of Ang-(1-7),^27^ (believed to be a potent anti-ischemic and cardioprotective agent),^12^ or increased angiogensis.^28^

Our findings do not chime in with previously reported clinical^29^ and epidemiological findings,^30^ suggesting that hypertension enhanced the cardiac complications of diabetes. The reason for such discrepancy might be due to the duration of such diseases, which is usually much longer in human than that in animal models. In the present study, the duration of diabetes was 10 weeks and that of renovascular hypertension was 4 weeks, whereas the duration of the development of such a disease are much longer, and almost all studies are unforthcoming as to how long the patients had the diseases before they entered the study. Moreover, our findings are not in agreement with previous studies indicating that DOCA-induced hypertension did not aggravate the severity of myocardial dysfunction in STZ-diabetic rats,^31^ hearts from diabetic hypertensive animals were less protected, hypertension deteriorated the cardiovascular complications of diabetes, and the complications of simultaneous hypertension plus diabetes were more severe than those of the diabetes alone.^8^^,^^10^ The difference between our findings and those of previous reports might be due to the differences in the animal model as well as the duration of diabetes and/or hypertension.

There are various models of experimental hypertension and type 2 diabetes. Each model of such diseases portrays a specific aspect of such diseases in humans, and none of them is a full representation of the diseases. It would be interesting to examine how the simultaneity of other models of hypertension and type 2 diabetes would affect cardiac functions. Whether or not the findings of the present study can be extended to a combination of other models and the significance of such findings needs further investigations. 

## Conclusion

The findings of the present study indicated that type 2 diabetes impaired cardiac functions, short-term renovascular hypertension improved cardiac functions, and simultaneity of hypertension with type 2 diabetes attenuated the diabetes-induced cardiac impairment. 
